# Sodium Reduction Program Incorporating Genetic Profile and an AI-Based App: A Randomized Clinical Trial

**DOI:** 10.1001/jamanetworkopen.2025.37540

**Published:** 2025-10-16

**Authors:** Koryu Sato, Kosuke Inoue, Taihei Yamaguchi, Maiko Yagi, Masashi Ebisawa, Shinichiro Mizuno, Tomoko Yanagisawa, Nana Akiyama, Koichi Node, Naoki Kondo, Toshi A. Furukawa

**Affiliations:** 1 Faculty of Policy Management, Keio University, Kanagawa, Japan; 2 Department of Social Epidemiology, Graduate School of Medicine and School of Public Health, Kyoto University, Kyoto, Japan; 3 Hakubi Center for Advanced Research, Kyoto University, Kyoto, Japan; 4 Innovation Laboratory, Next Business Development Division, Toshiba Corporation, Tokyo, Japan; 5 Department of Research & Development, Wellmira Inc, Tokyo, Japan; 6 Department of Clinical Genomics, The University of Tokyo Hospital, Tokyo, Japan; 7 Department of Cardiovascular Medicine, Saga University, Saga, Japan; 8 Japanese Society of Hypertension, Tokyo, Japan; 9 Office of Institutional Advancement and Communications, Kyoto University, Kyoto, Japan

## Abstract

**Importance:**

Excess salt intake is a major modifiable risk factor for hypertension and cardiovascular disease. Personalized interventions using genetic information and digital tools, such as smartphone applications, may enhance sodium reduction, but evidence remains limited.

**Objective:**

To evaluate the effectiveness of a sodium reduction program incorporating a genetic profile and an artificial intelligence (AI)–based mobile app among adults with elevated blood pressure and a sodium-sensitive genotype.

**Design, Setting, and Participants:**

A 3-arm randomized clinical trial, conducted at a Japanese electronics company from September 17 to December 16, 2024, included employees aged 20 to 65 years with elevated blood pressure and the sodium-sensitive *AGT M235T* genotype.

**Interventions:**

Participants were randomized on a 1:1:0.2 basis to (1) a treatment group receiving a genetic profile and an AI-based app incorporating sodium-specific information; (2) a control group receiving no intervention; or (3) an app-only group using the app without a genetic profile or information.

**Main Outcomes and Measures:**

The primary outcome was daily salt intake, estimated from spot urine samples using the INTERSALT (International Study of Electrolyte Excretion and Blood Pressure) formula. Secondary outcomes included self-reported body mass index, behavior change intentions, and systolic and diastolic blood pressure. Analysis was performed on an intention-to-treat basis.

**Results:**

Of 312 randomized participants, 289 completed follow-up (mean [SD] age, 51.3 [8.3] years; 252 of 279 men [90.3%]). The mean (SD) baseline salt intake was 11.3 (2.0) g/d. At 3 months, no significant difference in salt intake was observed between the treatment and control groups (mean difference, −0.2 g/d; 95% CI, −0.7 to 0.3 g/d) or between the treatment and app-only groups (mean difference, −0.04 g/d; 95% CI, −0.9 to 0.8 g/d). No significant differences were observed between groups in any secondary outcomes.

**Conclusions and Relevance:**

In this randomized clinical trial conducted among individuals with elevated blood pressure and a sodium-sensitive genotype, a sodium reduction program combining a genetic profile and an AI-based app with sodium-specific information did not significantly reduce salt intake. These findings highlight the challenges of achieving dietary behavior change through genetic personalized plus digital interventions.

**Trial Registration:**

http://umin.ac.jp/ctr Identifier: UMIN000052685

## Key Points

**Question:**

Does a sodium reduction program that incorporates a genetic profile and an artificial intelligence (AI)–based smartphone app effectively reduce salt intake among adults with elevated blood pressure and a sodium-sensitive genotype?

**Findings:**

In this randomized clinical trial of 289 participants, the intervention group did not show a statistically significant reduction in salt intake after 3 months compared with either the control group or the app-only group. Secondary outcomes, including body mass index, behavior change intentions, and blood pressure, also did not differ significantly between the groups.

**Meaning:**

A sodium reduction program integrating a genetic profile and an AI-based app did not result in meaningful dietary change among individuals with elevated blood pressure, suggesting limited utility of digital and genetic personalized interventions in modifying sodium-related health behaviors.

## Teaser

This randomized clinical trial evaluates the effectiveness of a sodium reduction program incorporating a genetic profile and an artificial intelligence (AI)–based mobile app at reducing salt intake among adults with elevated blood pressure and a sodium-sensitive genotype.

## Introduction

Excessive salt intake increases blood pressure and contributes to cardiovascular diseases in the general population.^1,2^ It is estimated that 3.2% of cardiovascular events and 3.9% of all deaths are attributable to excessive salt consumption.^3^ Many international guidelines recommend reducing salt intake for the general population, as the World Health Organization recommends an intake of less than 5 g/d.^4^ However, the response of blood pressure to salt intake varies between individuals, and it has been shown that several genetic polymorphisms modulate sodium sensitivity. The *M235T* polymorphism in the angiotensinogen (*AGT* [OMIM 106150.0001]) gene is associated with increased hepatic production of angiotensinogen, the precursor of angiotensin II, a potent vasoconstrictor.^5^ Elevated angiotensinogen levels enhance activity of the renin-angiotensin-aldosterone system, leading to sodium retention, vascular resistance, and ultimately higher blood pressure in sodium-sensitive individuals.^5,6,7,8^ “Precision nutrition” has attracted much attention in recent years, with an increasing number of attempts to provide tailored dietary advice based on an individual’s genetic profile.^9^ In particular, personalized approaches that take into account genetic polymorphisms related to sodium sensitivity could be an effective strategy for the prevention and management of hypertension.

Several personalized interventions based on genetic profiles have been implemented; however, their effectiveness remains inconclusive. A randomized clinical trial (RCT) in the US provided personalized gene-based dietary advice for the treatment group.^10^ After 12 months, the treatment group with sodium-sensitive genes exhibited significant reductions in salt intake if they received dietary recommendations after the provision of a genetic profile, whereas those without sodium-sensitive genes did not show differences compared with the control group, which received general dietary recommendations without a genetic profile. Another RCT conducted in 7 European countries evaluated various types of personalized nutrition advice based on individuals’ baseline diet, phenotype, and genotype.^11^ After a 6-month intervention, those who received personalized advice consumed less sodium than those in the control group, who received general dietary advice. However, the study found no evidence that including genotypic information enhanced the effectiveness of personalized advice.

Furthermore, smartphone applications (apps) may be useful for sodium reduction. For example, one app allows users to log their food intake and track nutrient consumption. In a 4-week RCT among healthy adults, participants using that app significantly reduced 24-hour urinary sodium excretion compared with those using paper diaries.^12^ Another intervention was a smartphone-based educational program delivered through primary schools in China, where children learned about sodium reduction and engaged their families in monitoring and lowering household salt intake. In a 12-month cluster RCT, the program significantly reduced salt intake and systolic blood pressure (SBP) in adults, but the effect on children was smaller and not statistically significant.^13^ Three apps allow users to scan grocery items and receive recommendations for lower-salt alternatives; RCTs for these apps did not detect a significant difference in 24-hour urinary sodium excretion.^14,15,16,17^ Given the diverse functionalities of sodium reduction apps and their varying effectiveness, further research is warranted.

In this study, we investigated the effectiveness of a sodium reduction program incorporating a genetic profile and an AI-based app. We hypothesized that providing individuals with personalized genetic risk information would increase perceived vulnerability and enhance motivation to change dietary behavior. When coupled with the AI-based app that delivers real-time, tailored dietary advice, this approach may address both the cognitive and practical barriers to sodium reduction that have limited the effectiveness of prior interventions. Participants with elevated blood pressure and the *AGT M235T* polymorphism were recruited from among employees of a Japanese electronics company. We focused on *AGT M235T* because it is the most prevalent polymorphism in the Japanese population among candidate sodium-sensitive polymorphisms. A previous study reported that the frequency of the *T235* allele is 81% among the Japanese population, which is much higher than seen in White individuals.^18^ After a 3-month intervention, daily salt intake was objectively assessed via spot urine collection. This study provides unique insights tailored to the cultural context of Japan, where traditional condiments such as soy sauce and miso contribute substantially to salt intake.

## Methods

### Study Design, Setting, and Participants

This 3-arm randomized clinical trial, conducted at a Japanese electronics company from September 17 to December 16, 2024, was approved by the ethics committees at Kyoto University and Toshiba Corp. As the study interventions were noninvasive and behavioral, no formal criteria for adverse events or harms were established. The trial protocol (Supplement 1) was preregistered on the University Hospital Medical Information Network (UMIN) Clinical Trials Registry (UMIN000052685).^19^ Data are available at the UMIN Individual Case Data Repository.^20^ All participants provided written or electronic informed consent before entering the trial. This report followed the Consolidated Standards of Reporting Trials (CONSORT) reporting guideline for RCTs.^21^

This study was conducted at Toshiba Corp, an electronics manufacturer in Japan. We recruited study participants from 11 605 employees who had already provided genetic samples to the company. Among the employees, 11 240 of 11 605 individuals (96.9%) carried at least 1 risk allele, in line with a previous study of the Japanese population.^18^ The inclusion criteria were as follows: (1) employees aged 20 to 65 years, (2) individuals carrying the *AGT M235T* polymorphism, (3) individuals with SBP of 120 mm Hg or higher or diastolic blood pressure (DBP) of 80 mm Hg or higher^22^ as indicated in health checkups conducted in 2022 or 2023, (4) individuals who had never used the AI-based app (see Intervention and Control Conditions for detailed information about the app), and (5) individuals who provided a spot urine sample and completed a questionnaire before the intervention. We excluded (1) individuals who were pregnant or breastfeeding, (2) individuals who did not own a smartphone (iOS or Android operating system) or were unable to use the app, and (3) individuals who could not understand Japanese.

### Randomization and Masking

Participants were randomized on a 1:1:0.2 basis to (1) the treatment group that underwent the sodium reduction program, (2) the control group that did not receive any intervention, or (3) the app-only group that did not receive a genetic profile and information on sodium reduction but was encouraged to use the app to estimate the specific contribution of the genetic information over and above the app use. The comparison of 1 vs 2 groups, therefore, answers the question of whether it is useful to add the intervention comprising the genetic information, app use, and sodium-specific information, while the comparison of 1 vs 3 groups estimates the specific effect of the intervention vs the app use per se. We set up the app-only group as a supplementary control group to investigate the effect of providing genetic profile and corresponding information, excluding that of the app use.

Randomization was conducted by designated Toshiba staff (T. Yamaguchi, M.Y., and M.E.). All interventions were delivered automatically, with no human involvement in the delivery process, thereby eliminating the need for blinding of intervention professionals. Due to the nature of the interventions, participants were aware of the content of the intervention they received. However, they were blinded to the difference between the groups and to the study hypothesis. Participants were told only that the study was comparing different methods for hypertension control. The difference between the groups was disclosed to participants only after study completion, per the ethics committee’s approval. Laboratory personnel responsible for measuring urinary sodium excretion were blinded to treatment allocation.

### Intervention and Control Conditions

Our sodium reduction program consisted of 3 components: (1) the provision of a genetic profile, (2) use of the AI-based app, and (3) the distribution of educational information on sodium reduction via the app. The treatment group was informed that they had a sodium-sensitive genotype via email on the first day of the intervention. The message explained that their blood pressure tends to be higher even with general salt intake, while sodium reduction in the daily diet is more effective in lowering blood pressure than for other people without the genotype (eMethods 1 in Supplement 2).

The AI-based health care app was developed by Wellmira Inc. Users record their daily meals, exercise, mood, sleep quality, and body weight on the app. The accuracy of the salt intake estimation based on recorded diet has been validated.^23^ For every user’s input, an AI program provides personalized advice (eMethods 2 in Supplement 2). The app has 11 programs tailored to the user’s objectives, with more than 200 million patterns of AI advice. The treatment group was instructed to select a lifestyle improvement course, which was developed under the supervision of diabetes specialists for those with a diagnosis of hypertension or diabetes.

The treatment group also received information on sodium reduction through the app twice a week. Twenty-seven articles were prepared under the supervision of a genetic information specialist (N.A.) and nutritionists (eMethods 3 in Supplement 2).

### Outcomes and Follow-Up

The primary outcome was salt intake, estimated from a spot urine sample using the INTERSALT (International Study of Electrolyte Excretion and Blood Pressure) formula (eMethods 4 in Supplement 2).^24^ Our secondary outcomes were self-reported body mass index (BMI), behavior change intentions, SBP, and DBP collected via an online survey. Behavior change intentions were conceptualized based on the transtheoretical model^25^ and assessed as a continuous variable using a single-item measure.^26^ The survey included an item asking “What do you think about improving eating habits?” with answer options on a 5-point Likert scale (1 = not interested, 2 = need to improve but cannot do it, 3 = want to do it now, 4 = have been implementing improvements for <6 months, or 5 = have been implementing improvements for >6 months).

At baseline, we collected participants’ information on age, sex, antihypertensive medication use, weight, and height to calculate BMI, SBP, and DBP from a health checkup conducted in 2023. A spot urine collection and an online survey measuring behavior change intentions were also conducted.

### Sample Size

To calculate the necessary sample size, we assumed an effect size of 0.7 g/d in salt intake reduction, as equivalent to a previous study.^11^ The expected SD of the salt intake estimation was set to 2.2 g/d, based on a systematic review.^27^ With a significance level of 5% and a statistical power of 90%, 374 participants were necessary. In addition, we planned to compare the treatment group with the app-only group, which did not receive a genetic profile or special information on sodium reduction but were encouraged to use the app as a supplementary analysis. To obtain a 95% CI of mean ±0.7 g/d, 31 participants would be necessary to be assigned to the app-only group, assuming an SD of 2.2 g/d. Expecting a 10% loss to follow-up, we originally aimed to recruit 451 employees in total.

### Statistical Analysis

The intention-to-treat effect was estimated using ordinary least squares by including binary variables indicating the app-only and control groups (hence, the treatment group is the reference). For sensitivity analysis, we adjusted for the baseline variables of age, sex, antihypertensive medication use, salt intake, BMI, behavior change intentions, SBP, and DBP. In addition, we performed the post hoc calculation of minimum detectable effect size, given the actual sample size and SD of salt intake. Observations with missing values were excluded from analyses. Statistical significance was defined as 2-tailed *P* < .05 without any corrections for multiple comparisons. Analyses were performed using R, version 4.4.1 (R Project for Statistical Computing) between February 13 and 14, 2025, by a researcher (K.S.) who was blinded to treatment allocation. The researcher was unblinded on February 18, 2025.

## Results

Of 312 randomized participants, 289 (92.6%) completed follow-up (Figure). Their mean (SD) age was 51.3 (8.3) years, 252 of 279 (90.3%) were men, and 27 of 279 (9.7%) were women (Table 1). The mean (SD) baseline salt intake was 11.3 (2.0) g/d. The employees were recruited from March 29 to July 31, 2024. Of 650 employees who consented, only 312 met the inclusion criteria. Nonetheless, 232 participants were sufficient for comparison between the treatment and control groups, given the lowered expectations for the SD of the salt intake estimation to 2.0 g/d and the statistical power to 80%, which is acceptable and common practice.^28,29^ Thus, participants were randomized and initiated the intervention. Of the eligible participants, 141 were assigned to the treatment group, 141 were assigned to the control group, and 30 were assigned to the app-only group. The intervention period was from September 17 to December 16, 2024. After the intervention, participants submitted their urinary specimens and follow-up surveys from December 2, 2024, to January 31, 2025 (2 participants sent a specimen before the end of the intervention period). The proportion of loss to follow-up was 7.8% (11 of 141) in the treatment group, 5.7% (8 of 141) in the control group, and 13.3% (4 of 30) in the app-only group. Participants who were lost to follow-up did not differ substantially in baseline characteristics compared with those who completed follow-up (eTable 1 in Supplement 2). Of the 289 participants who were followed up, 268 (92.7%) sent a urinary specimen, 276 (95.5%) reported their weight (BMI), 286 (99.0%) answered about behavior change intentions, and 128 (44.3%) reported SBP and DBP. We found no significant differences in baseline characteristics among those with a missing outcome (eTable 2 in Supplement 2). Four of 130 participants who were followed up in the treatment group did not download the app, while all participants in the app-only group downloaded it.

**Figure.**
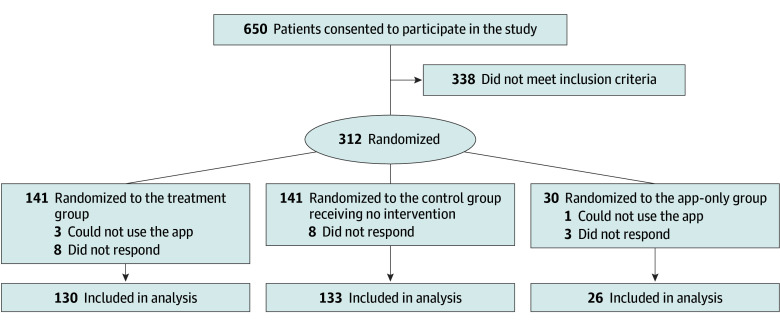
Study Flowchart

**Table 1.** Baseline Characteristics of Study Participants

Characteristic	Overall (N = 289)	Treatment group (n = 130)	Control group (n = 133)	App-only group (n = 26)
Age, mean (SD), y^a^	51.3 (8.3)	51.5 (8.0)	51.2 (8.4)	51.1 (9.6)
Sex, No./total No. (%)^a^				
Men	252/279 (90.3)	112/125 (89.6)	116/129 (89.9)	24/25 (96.0)
Women	27/279 (9.7)	13/125 (10.4)	13/129 (10.1)	1/25 (4.0)
Antihypertensive drug (yes), No./total No. (%)	153/289 (52.9)	65/130 (50.0)	70/133 (52.6)	18/26 (69.2)
Salt intake, mean (SD), g/d^a^	11.3 (2.0)	11.4 (2.0)	11.2 (2.0)	11.5 (2.2)
BMI, mean (SD)^a^	25.3 (4.0)	25.4 (4.0)	25.3 (4.0)	24.6 (4.2)
Behavior change intentions, mean (SD)	2.7 (1.2)	2.7 (1.2)	2.7 (1.2)	2.9 (1.3)
SBP, mean (SD), mm Hg^a^	127.8 (12.4)	128.0 (11.6)	127.6 (13.4)	127.8 (11.3)
DBP, mean (SD), mm Hg^a^	81.3 (10.5)	82.4 (9.6)	81.1 (9.3)	77.0 (18.0)

Abbreviations: BMI, body mass index (calculated as weight in kilograms divided by height in meters squared); DBP, diastolic blood pressure; SBP, systolic blood pressure.

^a^ Data from the health checkup in 2023 is missing for 10 participants (5 in the treatment group, 4 in the control group, and 1 in the app-only group). Hence, age, sex, salt intake (estimated based on age, sex, and BMI), BMI, SBP, and DBP include 10 missing values.

At 3 months, the treatment group did not show a significant reduction in salt intake compared with the control group (mean difference, −0.2 g/d; 95% CI, −0.7 to 0.3 g/d) or the app-only group (mean difference, −0.04 g/d; 95% CI, −0.9 to 0.8 g/d) (Table 2). In addition, there were no significant differences between groups in BMI, behavior change intentions, SBP, and DBP.

**Table 2.** Mean Values and Between-Group Differences in Outcomes at 3 Months

Outcome	No.	Mean (SD) value			Difference (95% CI)	
		Treatment group	Control group	App-only group	Treatment vs control	Treatment vs app only
Salt intake, g/d	268	10.8 (2.0)	11.0 (2.0)	10.9 (2.1)	−0.2 (−0.7 to 0.3)	−0.04 (−0.9 to 0.8)
BMI	276	25.5 (4.6)	25.5 (4.0)	24.9 (4.1)	0.1 (−1.0 to 1.1)	0.7 (−1.2 to 2.5)
Behavior change intentions	286	2.9 (1.2)	2.7 (1.1)	3.0 (1.2)	0.2 (−0.1 to 0.4)	−0.1 (−0.6 to 0.4)
SBP, mm Hg	128	130.2 (19.9)	132.8 (11.5)	134.2 (12.9)	−2.6 (−8.7 to 3.4)	−4.1 (−13.1 to 4.9)
DBP, mm Hg	128	83.4 (14.6)	85.7 (8.3)	88.3 (9.7)	−2.3 (−6.7 to 2.1)	−4.9 (−11.5 to 1.7)

Abbreviations: BMI, body mass index (calculated as weight in kilograms divided by height in meters squared); DBP, diastolic blood pressure; SBP, systolic blood pressure.

For the sensitivity analysis, adjustments were made for the baseline variables, and consistent results were found (eTable 3 in Supplement 2). No significant differences were observed, except for DBP between the treatment and app-only groups (mean difference, −9.2 mm Hg; 95% CI, −16.0 to −2.4 mm Hg). The minimum detectable effect sizes were 0.7 g/d with a power of 80% and 0.8 g/d with a power of 90% (eMethods 5 in Supplement 2).

## Discussion

In this RCT, we evaluated the effectiveness of a sodium reduction program that integrated a genetic profile and an AI-based app among Japanese employees with elevated blood pressure and a sodium-sensitive genotype. After a 3-month intervention, no statistically significant reduction in salt intake was observed in the treatment group compared with the control or app-only groups. Secondary outcomes, including BMI, behavior change intentions, and blood pressure, also showed no significant differences between groups.

Our null results contribute to the ongoing discourse on the effectiveness of personalized dietary interventions. Several factors may explain the lack of effectiveness in our intervention. First, the effect of receiving a genetic profile on behavior change may be limited, as we showed that the scores of the behavior modification stage did not change. Our findings align with meta-analyses that have questioned the behavioral benefits of genetic risk communication.^30,31^ Moreover, the mode and frequency of delivering genetic profiles might have influenced its effectiveness. In our study, a genetic profile was provided via a single email at the beginning of the intervention. A single exposure to a genetic profile may have been insufficient to sustain behavior change. Second, user engagement with the app may have been suboptimal. The effectiveness of app-based interventions relies heavily on consistent and active user participation. A 3-month RCT evaluated the effectiveness of the AI-based app in our present study on weight reduction among adults with overweight or obesity and demonstrated significant weight loss.^32^ In the previous study, the study staff checked participants’ input and emailed them once a week to encourage input if they had recorded diet information less than 4 days per week. In the present study, however, we did not prompt participants to continue app use even when they discontinued meal logging because we intended to evaluate the effectiveness of the intervention without human support. Insufficient engagement with the app could have diminished its efficacy. Third, our findings suggest the difficulty of achieving behavioral change among middle-aged men (the sample was 90.3% men, with a mean [SD] age of 51.3 [8.3] years) with elevated blood pressure. Several studies suggest that middle-aged men with hypertension face significant barriers to behavior change, including low disease awareness, cultural dietary preferences, limited self-management skills, and competing responsibilities such as work and family.^33,34^ Our sodium reduction program was not specifically designed for this demographic group and may result in a limited effect. Furthermore, there were notable differences in food culture between previous studies and our study. Japanese cuisine often includes high-sodium foods, such as miso soup, pickled vegetables, and soy sauce–based dishes, making sodium reduction challenging.

### Limitations

This study has several limitations. First, the generalizability of our findings is limited to middle-aged, predominantly male employees of a single Japanese corporation. Demographic differences in health behavior change, caregiving responsibilities, and technology use may affect engagement and responsiveness to genetic information and digital health tools. Future studies should evaluate whether similar interventions are effective in more diverse populations, including retired individuals or women. Second, while spot urine collection is a more objective method than self-report questionnaires, it is not equivalent to 24-hour urine collection, the criterion standard for salt intake assessment. The use of estimation formulas may introduce biases, although standardized protocols were used to mitigate this. Third, the small sample size and the short follow-up duration might explain the null results. However, our study had a larger sample size than most existing RCTs and a comparable follow-up duration.^35^ In addition, the post hoc calculation of minimum detectable effect size suggested that our study was sufficiently powered to detect moderate differences as small as 0.35 to 0.40 SD. Fourth, we did not quantitatively track app engagement metrics due to participant privacy concerns. Understanding the extent and patterns of app use would have provided valuable insights into the association between engagement and outcomes. Fifth, because all participants were employees of the same company, informal discussions about the study could have occurred despite our efforts to conceal allocation and deliver all materials individually. Any exchange of information, particularly disclosure of app features or genetic-risk feedback, might have attenuated true between-group differences and biased results toward the null.

## Conclusions

In this RCT of a sodium reduction program incorporating a genetic profile and an AI-based app among adults with elevated blood pressure and a sodium-sensitive genotype, we did not find evidence supporting its effectiveness. Despite technological advancements and the promise of personalized nutrition, our findings highlight the challenges of translating genetic and digital interventions into meaningful dietary behavior change.

## Notes

Supplement 1.
Trial Protocol and Statistical Analysis Plan

Supplement 2. **eMethods 1.** The Text of the Genetic Information Disclosure E-Mail **eMethods 2.** Images of Recording Diet With the App **eMethods 3.** Example of Educational Information on Sodium Reduction **eMethods 4.** The INTERSALT Formula **eTable 1.** Comparison of Baseline Characteristics Between Followed and Lost-to-Follow-Up Participants **eTable 2.** Comparison of Baseline Characteristics of Participants With Missing Outcomes **eTable 3.** Baseline Adjusted Mean Scores and Between-Group Differences in Outcomes at 3 Months **eMethods 5.** Post Hoc Calculation of Minimum Detectable Effect Size (MDES)

Supplement 3.
Data Sharing Statement
