# Supplementary material for: Sodium Reduction Program Incorporating Genetic Profile and an AI-Based App: A Randomized Clinical Trial
Source: JAMA Netw Open. 2025 Oct 16;8(10):e2537540. doi: 10.1001/jamanetworkopen.2025.37540 (PMC12531883; doi:10.1001/jamanetworkopen.2025.37540)
Supplement: Supplement 1. — Trial Protocol and Statistical Analysis Plan [file jamanetwopen-e2537540-s001.pdf]

Study Protocol

1. Title of the Study

Randomized Controlled Trial of an AI-based Health App Using Genetic Information in Individuals with Elevated Blood Pressure

2. Background

Excessive salt intake is a primary cause of hypertension, yet individuals exhibit different degrees of salt sensitivity, i.e., the blood pressure response to salt intake. Salt sensitivity is known to be influenced by certain genetic variants.[1,2] Therefore, identifying individuals with high salt sensitivity genotypes and providing them with personalized, gene-informed guidance may yield substantial health benefits.[3] However, the evidence on the effectiveness of such interventions remains inconclusive.

Nielsen & El-Sohemy (2014) reported that a group receiving personalized advice including genetic information via monthly emails had reduced salt intake after 12 months compared to those receiving generic advice.[4] Celis-Morales et al. (2017) found that personalized online advice delivered every three months led to reduced salt intake after 6 months, but incorporating genetic information did not significantly increase the effect.[5] These interventions were not tailored to salt reduction and were conducted among general adults with relatively infrequent contact.

3. Objective and Significance

1）Objective

The objective of this study is to determine the effect of notifying individuals with salt sensitivity genotypes and delivering personalized dietary advice through the AI-based health app "*CALOmama PLUS*" on salt intake in individuals with elevated blood pressure.

2）Social Significance

The social significance of the study lies in the feasibility of identifying salt-sensitive genotypes through blood tests and the low-cost, high-frequency nature of app-based interventions. If the combination of genetic testing and app-based personalized dietary guidance effectively reduces salt intake, it may provide an efficient strategy to prevent hypertension.

4. Participant Selection

1) Setting: Employees of Toshiba Corporation

2) Inclusion Criteria
- Age 20–65
- Participants in Toshiba's genome and health data integration project, specifically those with AGT M235T genotype
- Systolic blood pressure ≥120 mmHg or diastolic blood pressure ≥80 mmHg in 2022 or 2023 health checkups
- No prior use of *CALOmama PLUS*
- Submitted spot urine sample and completed the pre-intervention survey

3) Exclusion Criteria
- Pregnant or lactating
- No smartphone (iPhone or Android)
- Cannot understand written Japanese

- Deemed ineligible by study investigators

4) Sample Size and Rationale

A total of 451 participants are planned, with 208 allocated to the intervention group, 208 to the control group, and 35 to the app-only group. Celis-Morales et al. (2017) reported a 0.74g reduction in salt intake after 3 months in the intervention group compared to the control group.[5] In addition, a systematic review by Huang et al. (2016)[7] found that the standard deviation (SD) of estimated salt intake using the INTERSALT method[8] from spot urine samples ranged from approximately 2.0 to 2.2g. Assuming an effect size of 0.74g and an SD of 2.2g, with a significance level of 5% and power of 90%, the required sample size for the primary comparison between the intervention group and the control group is 187 participants per group, totaling 374 participants. For the supplementary analysis comparing the intervention group and the app-only group, a sample size of 31 is required to obtain a 95% confidence interval of ±0.74g, assuming the same SD and significance level. Adding these together results in 405 participants, and allowing for a maximum dropout rate of 10%, the final sample size is set at 451 participants.

5. Methods and Scientific Validity

1) Study Design

This study is a three-arm parallel-group randomized controlled trial. Participants in the intervention group will be informed at the beginning of the trial that they carry a salt sensitivity genotype and will be asked to record their dietary intake using the *CALOmama PLUS* app. They will also receive weekly columns and quizzes related to salt reduction via the app. The control group will receive no intervention. The app-only group will be asked to record their dietary intake using the *CALOmama PLUS* app, but they will not be informed of their genetic information, nor will they receive any salt reduction content. Of the 451 study participants, 208 will be randomly allocated to the intervention group, 208 to the control group, and 35 to the app-only group. Over 600 individuals have already applied, ensuring the target sample size is attainable. Participants will not be informed of their group allocation (participant blinding). Evaluators at Kyoto University will also be blinded to group assignments (evaluator blinding).

	Control group (n = 208)	App-only group (n = 35)	Intervention group (n = 208)
Genetic profile	×	×	✓
CALOmama PLUS app use	×	✓	✓
Information on sodium reduction	×	×	✓

2) Methods

Toshiba Corporation will recruit volunteers from among its employees and obtain informed consent via electronic signature through a dedicated website. Each consenting participant will be automatically assigned a study ID. Toshiba will verify that the consenting individuals are valid employees with internal email addresses, confirm their genotype and blood pressure, and notify eligible individuals of their study ID and the URL for the urine test.

Spot urine analysis will be conducted by Healthcare Systems Co., Ltd. (HS), commissioned by Wellmira Inc. Participants will input their ID, name, address, email address, healthcare utilization status, and health awareness responses into a form accessed via the URL provided by Toshiba. HS will mail test kits for both pre- and post-intervention collection and conduct the initial urine analysis. HS will report the ID, email address, health awareness and medical visit survey responses, and test results to Wellmira. Wellmira will then report the ID, health awareness and medical visit responses, and urine submission status to Toshiba.

Toshiba will finalize participants based on urine submission and survey completion, and randomly assign them into the three groups. Toshiba will generate a participant list with IDs, allocation groups, and health check data from 2023 (sex, age, height, weight, systolic and diastolic blood pressure) and provide this to Wellmira.

Wellmira will inform participants in the intervention group that they carry a salt sensitivity genotype and request that they log every meal in the *CALOmama PLUS* app. Weekly columns and quizzes on salt sensitivity and salt reduction will be delivered via the app. Participants in control group 2 will be asked to record their meals using the app but will not receive genetic information or salt reduction content. The intervention period using *CALOmama PLUS* will last for 3 months.

At the end of the trial, Toshiba and Wellmira will instruct participants to submit post-intervention urine samples using the pre-mailed kits and complete a follow-up survey via a URL. Information collected will include ID, health awareness, age, weight, systolic and diastolic blood pressure (if measurable), and medical visit status. HS will report post-intervention urine test results to Wellmira and notify participants of their own pre- and post-test results.

Wellmira will provide Kyoto University with anonymized data including participant ID, allocation group, sex, pre- and post-intervention urine test results, health awareness, age, height, weight, blood pressure, and medical visit status. Group labels will be coded as Group A, B, or C to maintain evaluator blinding. Once the analysis is complete and results are confirmed, evaluator blinding will be lifted—no later than two months after data delivery. Toshiba recruits participants and obtains e-consent. Participants are screened for eligibility and randomized into three groups. Spot urine samples are analyzed by Healthcare Systems Co., Ltd. under contract from Wellmira Inc. Health behavior and awareness surveys are conducted online. Wellmira manages intervention content and data anonymization. Kyoto University conducts analysis in a blinded manner.

3) Measures and Schedule

i. Measurement items, methods, and agencies

a. Salt intake before and after the intervention will be measured by HS, commissioned by Wellmira Inc., using spot urine tests. Sodium, creatinine, and potassium excretion will be analyzed. Kyoto University will estimate daily salt intake using the INTERSALT formula[8] adjusted for BMI and age. If samples are not submitted by the deadline, Wellmira will follow up with participants.
b. Behavior change intentions and healthcare utilization before and after the intervention will be assessed via online surveys based on the transtheoretical model.[9] The question will be “What do you think about improving eating habits?” with options: 1 = “Not interested”, 2 = “Need to improve but cannot do it”, 3 = “Want to do it now”, 4 = “Have been implementing improvements for less than 6 months”, or 5 = “Have been implementing improvements for more than 6 months”. Medical visit status will be surveyed similarly. If responses are not submitted on time, Wellmira will follow up. Pre-intervention surveys will be administered by HS, post-intervention by Wellmira.
c. Post-intervention age, weight, and systolic/diastolic BP will be collected via online survey by Wellmira. If values differ greatly from baseline health check data, Wellmira will contact participants for confirmation. Reminders will be sent for unsubmitted surveys.
d. Pre-intervention age, sex, height, weight, and blood pressure will be provided by Toshiba

based on 2023 health check data.

ii. Measurement Schedule

Date	Events
March 29, 2024-July 3, 2024	Obtain consent for participation
July 30, 2024	Submit spot urine and complete survey (approx. 1 minute)
September 10, 2024<br>(start of trial)	Intervention group: Notified of salt-sensitive genotype and asked to log meals in *CALOmama PLUS* for 3 months<br>Control group: No action<br>App-only group: Asked to log meals in *CALOmama PLUS* for 3 months
December 9, 2024<br>(end of trial)	All participants: Submit spot urine and complete survey (approx. 1 minute)<br>Control and app-only groups: Notified of salt-sensitive genotype<br>Control group: May begin using *CALOmama PLUS* if desired

4) Analysis Overview

The primary analysis will compare the intervention group to the control group. A supplementary analysis will compare the intervention group to the app-only group to assess the effect of *CALOmama PLUS* use alone.

i. Primary Outcome: Sodium intake
ii. Secondary Outcomes: Behavior change intentions, systolic and diastolic blood pressure, BMI
iii. Main Analytical Method: Analysis of covariance

6. Study Duration

1） Recruitment Period
From the date of institutional approval to July 3, 2024

2） Intervention Period
Three months from the date of recruitment

3） Study Period
From the date of institutional approval to March 31, 2026

7. Informed Consent

Toshiba Corporation will set up a dedicated website accessible only to its employees, through which it will provide explanations about the study and obtain electronic signatures on the informed consent form from interested participants. The company will offer opportunities for participants to ask questions about the study content and will provide adequate answers. After obtaining informed consent, participants will have continued access to the consent materials. Printed documents will be provided upon request.

8. Handling of Personal Information

1） Types of Personal Information Collected
Personal information

2） Creation Period and Method
In July 2024, when participants are finalized, Toshiba will generate an ID list electronically. This list will be managed and stored by individuals within Toshiba who are not involved in this study, and study personnel will not have access to it.

3） Types of Personal Information Retained and Security Measures

Each institution will manage personal data as follows. Data will be encrypted with passwords and either transported on secure hard drives or transferred via encrypted file transmission systems. All data will be analyzed on computers equipped with security software, stored in physically locked rooms.

	Toshiba	Wellmira	HS	Kyoto Univ.
Name/Address	×	×	✓	×
Age/Sex	✓	✓	✓	✓
Study ID	✓	✓	✓	✓
Email	×	✓	✓	×
Health Checkup	✓	✓	×	✓
Urine Results	×	✓	✓	✓
Survey Results	×	✓	✓	✓

4） Data Management Responsibility

Department of Social Epidemiology, Graduate School of Medicine and School of Public Health, Kyoto University

Koryu Sato (part-time researcher)

5） Handling of Data Upon Withdrawal of Consent

If a participant withdraws consent before data analysis begins, Wellmira will delete the relevant data and not provide it to Kyoto University. If data has already been transferred, Kyoto University will delete it. Once data analysis is complete and blinding is lifted, consent cannot be withdrawn. This is clearly stated in the consent form.

9. Risks, Burdens, and Benefits

1） Burdens and Risks

Participants will submit spot urine samples and complete surveys before and after the intervention. Those in the intervention and app-only groups will be asked to log meals using the *CALOmama PLUS*. Additionally, being informed of one's salt-sensitive genotype may cause anxiety.

2） Benefits

Participants will receive notifications of their estimated salt intake after the study. Those in the intervention group will learn their genotype at the start, while participants in the control and app-only groups will be informed after the trial. All participants will eventually have access to the *CALOmama PLUS* app and AI-based dietary advice.

3） Overall Risk-Benefit Assessment

This non-invasive study is considered low burden, especially in comparison to prior similar studies.[4,5]

4） Risk Minimization Measures

Questions about genetic results will be answered based on physician-supervised guidance. Participants needing further support will be referred to the Department of Genetic Medicine at Kyoto University Hospital.

10. Sample and Data Storage and Disposal

1) Storage Duration

Samples and data will be stored for 10 years after the study concludes, in accordance with institutional policies.

2) Storage Method

i. Toshiba

Data will be stored on electronic media, in a locked safe installed in a security-enhanced area of the company equipped with surveillance cameras. Entry to this area is restricted by biometric authentication and electronic locks to prevent access by unauthorized individuals. For analysis purposes, a dedicated analysis computer located within this secure area will be used in a completely offline (disconnected) state. The analysis computer will be periodically connected to internal servers protected by the company's firewall to update antivirus software and operating system patches, managed by the administrator.

ii. Wellmira

Personal data will be stored on Wellmira's dedicated internal server for personal information. Access to this server is strictly limited to the designated personal information manager and a small number of authorized personnel within the company. The server is located in a security room equipped with surveillance cameras and electronic locks to control entry and exit, managed under the supervision of the personal information manager. The server is also regularly connected to internal management servers within the firewall for antivirus and OS updates. To respond to inquiries, the research-specific email addresses, group allocation information (intervention, control group, app-only group), and participant IDs are temporarily stored. Additionally, the post-trial survey data, which is collected via Google Forms, will be temporarily stored on Google Workspace cloud storage. After the response deadline, the survey form will be immediately transferred to Wellmira's secure internal server, and all data will be permanently deleted from the cloud. Any information received from Toshiba or Healthcare Systems Co., Ltd. that is encrypted with passwords—whether transported via hard drive or file transmission systems—will also be transferred to the secure server. Urine samples collected by HS on behalf of Wellmira will not be stored after measurement and will be immediately discarded.

iii. Kyoto University

Data will be stored on a computer installed with security software. This computer will be located in a physically locked room to prevent unauthorized access.

3) Post-Retention Disposal

Data will be permanently destroyed using irreversible methods at separate locations.

4) Data Transfer and Logging

When data is shared between institutions, detailed records of the transaction, including dates, recipient institutions, responsible persons, and data types, will be maintained and stored securely.

11. Secondary Use and Data Sharing

Study data may be shared with researchers, public health authorities, and other non-profit entities for medical or scientific advancement, subject to agreement among Kyoto University, Toshiba, and Wellmira.

Ethics review will be required prior to any secondary use. Participants will be notified by email and may opt out.

12. Reporting to Ethics Committee and Institutional Officials

Any information that may compromise scientific validity or ethical appropriateness will be promptly reported. Annual progress reports and final study reports will be submitted as required.

13. Funding and Conflicts of Interest

1） Funding Source

This study is funded by the Japan Agency for Medical Research and Development (AMED) under the program “Research Infrastructure Development for Implementation of Preventive and Health Promotion Measures in Society.” Wellmira Inc. is a research collaborator under this AMED program and does not provide direct financial support. However, costs associated with this study—including provision of the *CALOmama PLUS* app, personnel expenses for labor, and outsourcing fees for urine testing—are covered by the AMED project. Toshiba Corporation is also a participating institution in the AMED project and does not provide financial support; however, it contributes in-kind by providing labor such as participant recruitment and data provision free of charge.

2） Sponsor-Investigator Relationships

AMED is not involved in the design, conduct, analysis, or publication of the study. Toshiba and Wellmira are co-investigators and will be listed as co-authors.

3） Conflicts of Interest

Koryu Sato has received compensation from Wellmira for unrelated advisory services. Conflicts of interest have been reviewed by Kyoto University’s COI Committee. Each institution applies its own regulations accordingly.

14. Public Disclosure of Study Information

The study has been registered with the UMIN Clinical Trials Registry (UMIN000052685). Results will be presented in academic conferences and peer-reviewed journals. Pseudonymized data will be published on UMIN-ICDR.

15. Participant and Public Inquiries

1） Contact for Study-Related Inquiries

Toshiba Corporation
Innovation Laboratory, Next Business Development Division
(Tel) +81-3-3457-2984 (Weekdays 10:00–17:00)
(E-mail) HdqPM-GenomePJ@ml.toshiba.co.jp

Contact for Inquiries about the AI Health App

Wellmira Inc.
(E-mail) kawarunavi@linkncom.co.jp (Weekdays 10:00–17:00)

Department of Social Epidemiology, Graduate School of Medicine and School of Public Health, Kyoto University
(Tel) +81-75-753-4355 (Weekdays 10:00–17:00)

2） Contact for Complaints or General Inquiries at Kyoto University

General Affairs and Planning Division, Research Promotion Section, Graduate School of Medicine and School of Public Health, Kyoto University
(Tel) +81-75-753-9301
(E-mail) 060kensui@mail2.adm.kyoto-u.ac.jp

Contact for Genetic Counseling
Department of Genetic Medicine, Kyoto University Hospital
(Tel) +81-75-751-4350 (Weekdays 13:00–16:30)

16. Participant Compensation and Cost Burden

1） Compensation

None.

2） Participant Costs

None.

17. Feedback of Study Results to Participants

Participants will receive information about their salt intake estimates based on pre- and post-intervention urine samples.

18. Study Governance

1） Principal Investigator

Kosuke Inoue
Associate Professor, Department of Social Epidemiology, Graduate School of Medicine and School of Public Health, Kyoto University (Overall study coordination, planning, and manuscript preparation)

2） Co-Investigators

Toshi A. Furukawa
Professor, Office of Institutional Advancement and Communications, Kyoto University (Planning, and manuscript preparation)

Koryu Sato
Part-Time Researcher, Department of Social Epidemiology, Graduate School of Medicine and School of Public Health, Kyoto University (Planning, study implementation, data analysis, and manuscript preparation)

3） Collaborating Institutions

Taihei Yamaguchi
Director, Innovation Laboratory, Next Business Development Division, Toshiba Corporation

Masayuki Irisawa
Chief Productivity Officer, Wellmira Inc.

4） Urine Analysis Contractor

Yosuke Takimoro
Chief Executive Officer, Healthcare Systems Co., Ltd.

19. Protocol Revisions

Amendments to the study protocol must be reviewed and approved by the ethics committee.

20. Ethical Standards

The study will comply with the Declaration of Helsinki and national ethics guidelines for biomedical research involving human subjects. Ethics approval has been obtained from the Kyoto University Graduate School and Faculty of Medicine Ethics Committee.

21. Ownership of Study Results

The study results will be jointly owned by Kyoto University, Toshiba Corporation, and Wellmira Inc.

22. References

1. Hunt SC, Cook NR, Oberman A, et al. Angiotensinogen genotype, sodium reduction, weight loss, and prevention of hypertension: trials of hypertension prevention, phase II. *Hypertens Dallas Tex 1979*. 1998;32(3):393-401. doi:10.1161/01.hyp.32.3.393
2. Norat T, Bowman R, Luben R, et al. Blood pressure and interactions between the angiotensin polymorphism AGT M235T and sodium intake: a cross-sectional population study. *Am J Clin Nutr*. 2008;88(2):392-397. doi:10.1093/ajcn/88.2.392
3. Svetkey LP, Moore TJ, Simons-Morton DG, et al. Angiotensinogen genotype and blood pressure response in the Dietary Approaches to Stop Hypertension (DASH) study. *J Hypertens*. 2001;19(11):1949-1956. doi:10.1097/00004872-200111000-00004
4. Nielsen DE, El-Sohemy A. Disclosure of genetic information and change in dietary intake: a randomized controlled trial. *PloS One*. 2014;9(11):e112665. doi:10.1371/journal.pone.0112665
5. Celis-Morales C, Livingstone KM, Marsaux CF, et al. Effect of personalized nutrition on health-related behaviour change: evidence from the Food4Me European randomized controlled trial. *Int J Epidemiol*. 2017;46(2):578-588. doi:10.1093/ije/dyw186
6. American Heart Association. Understanding Blood Pressure Readings. American Heart Association. Published May 2023. Accessed November 2, 2023. https://www.heart.org/en/health-topics/high-blood-pressure/understanding-blood-pressure-readings
7. Huang L, Crino M, Wu JHY, et al. Mean population salt intake estimated from 24-h urine samples and spot urine samples: a systematic review and meta-analysis. *Int J Epidemiol*. 2016;45(1):239-250. doi:10.1093/ije/dyv313
8. Brown IJ, Dyer AR, Chan Q, et al. Estimating 24-hour urinary sodium excretion from casual urinary sodium concentrations in Western populations: the INTERSALT study. *Am J Epidemiol*. 2013;177(11):1180-1192. doi:10.1093/aje/kwt066
9. Prochaska JO, DiClemente CC. The transtheoretical approach. In: *Handbook of Psychotherapy Integration, 2nd Ed*. Oxford series in clinical psychology. Oxford University Press; 2005:147-171. doi:10.1093/med:psych/9780195165791.003.0007
