# Supplementary material for: Sodium Reduction Program Incorporating Genetic Profile and an AI-Based App: A Randomized Clinical Trial
Source: JAMA Netw Open. 2025 Oct 16;8(10):e2537540. doi: 10.1001/jamanetworkopen.2025.37540 (PMC12531883; doi:10.1001/jamanetworkopen.2025.37540)
Supplement: Supplement 2. — eMethods 1. The Text of the Genetic Information Disclosure E-Mail eMethods 2. Images of Recording Diet With the App eMethods 3. Example of Educational Information on Sodium Reduction eMethods 4. The INTERSALT Formula eTable 1. Comparison of Baseline Characteristics Between Followed and Lost-to-Follow-Up Participants eTable 2. Comparison of Baseline Characteristics of Participants With Missing Outcomes eTable 3. Baseline Adjusted Mean Scores and Between-Group Differences in Outcomes at 3 Months eMethods 5. Post Hoc Calculation of Minimum Detectable Effect Size (MDES) [file jamanetwopen-e2537540-s002.pdf]

Supplementary Online Content

Sato K, Inoue K, Yamaguchi T, et al. Sodium reduction program incorporating genetic profile and an AI-based app: a randomized clinical trial. *JAMA Netw Open.* 2025;8(10):e2537540. doi:10.1001/jamanetworkopen.2025.37540

eMethods 1. The Text of the Genetic Information Disclosure E-Mail

eMethods 2. Images of Recording Diet With the App

eMethods 3. Example of Educational Information on Sodium Reduction

eMethods 4. The INTERSALT Formula

eTable 1. Comparison of Baseline Characteristics Between Followed and Lost-to-Follow-Up Participants

eTable 2. Comparison of Baseline Characteristics of Participants With Missing Outcomes

eTable 3. Baseline Adjusted Mean Scores and Between-Group Differences in Outcomes at 3 Months

eMethods 5. Post Hoc Calculation of Minimum Detectable Effect Size (MDES)

This supplementary material has been provided by the authors to give readers additional information about their work.

eMethods 1. The Text of the Genetic Information Disclosure E-Mail

You have been found to have a genetic polymorphism (constitution) that makes you highly sodium-sensitive. It is known that if you have this constitution, your blood pressure tends to be higher even with a general salt intake. On the other hand, it is also known that reducing the amount of salt in the diet (sodium reduction) is more effective in lowering blood pressure than in other people.

Two factors influence the development of hypertension: genetic factors, which are the body constitution as revealed in this study, and environmental factors such as salt intake. To prevent high blood pressure, it is important to reduce salt intake from the daily diet (sodium reduction).

© 2025 Sato K et al. *JAMA Network Open.*

eMethods 2. Images of Recording Diet With the App

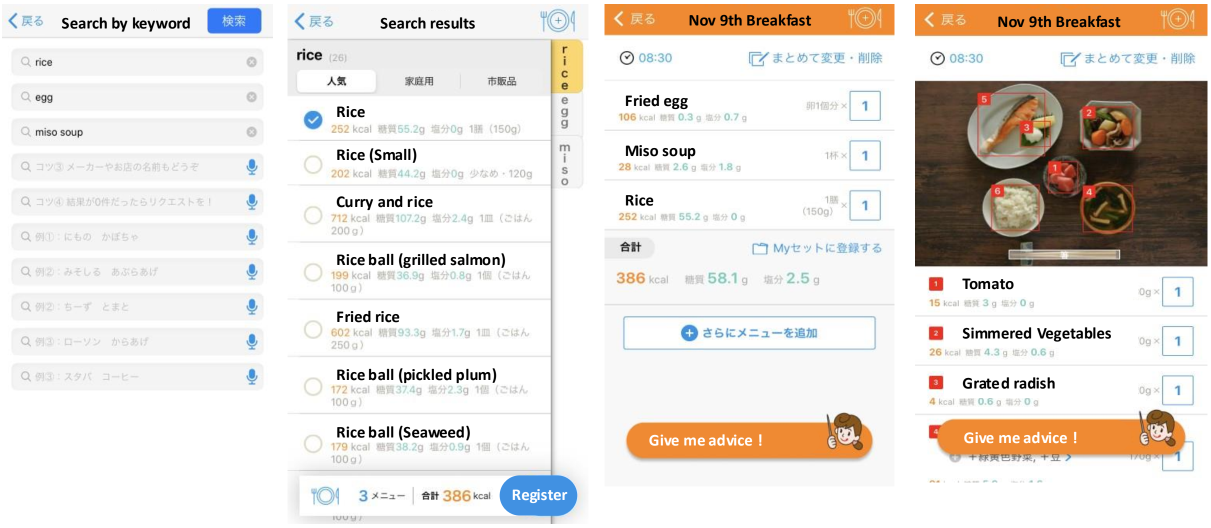

Note: Users can search for food and menu by keyword retrieval. Meals and their nutritional contents are stored in the database of the app. Artificial intelligence supports accurate recording by detecting photos of the meals taken by the users. © Wellmira Inc. Used with permission.

eMethods 3. Example of Educational Information on Sodium Reduction

Excess Salt Intake from Soups

Soups are a staple in daily meals, but they often contain more than 1g of salt per serving. Some noodle dishes can even exceed the daily recommended salt intake (7.5g for men, 6.5g for women) in just one meal. Here are some tips to help reduce salt intake while still enjoying soups!

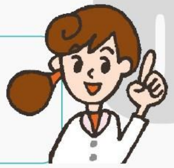

Tips for Reducing Salt in Soups

Increase the amount of ingredients

Adding more vegetables, mushrooms, and seaweed can help reduce the amount of broth needed while also increasing nutrient intake—a win-win!

Leave some broth behind

When eating ramen, soba, or udon, drinking all the broth can lead to excessive salt intake. Eat slowly and try to leave some broth behind.

Utilize umami and broth-based

Ingredients like garlic, onions, and tomatoes add natural umami flavor. Use seasonings like dashi-blended soy sauce or ponzu sauce instead of regular soy sauce for a flavorful yet lower-sodium option.

Note: © Wellmira Inc. Used with permission.

eMethods 4. The INTERSALT Formula

Men: salt intake (g/day) = [25.46 + 0.46 × spot Na (mmol/L) – 2.75 × 0.00884 × spot Cr (mg/dL) – 0.13 × spot K (mmol/L) + 4.10 × BMI (kg/m^2) + 0.26 × age (year)] × 23 × 2.54 / 1000

Women: salt intake (g/day) = [5.07 + 0.34 × spot Na (mmol/L) – 2.16 × 0.00884 × spot Cr (mg/dL) – 0.09 × spot K (mmol/L) + 2.39 × BMI (kg/m^2) + 2.35 × age (year) – 0.03 × age^2 (year)] × 23 × 2.54 / 1000

© 2025 Sato K et al. *JAMA Network Open.*

eTable 1. Comparison of Baseline Characteristics Between Followed and Lost-to-Follow-up Participants

**Characteristic**	**Followed Up**	**Lost to Follow-up**		
	**Overall**, N = 289	**Treatment**, N = 11	**Control**, N = 8	**App only**, N = 4
Age (year), Mean (SD)	51.3 (8.3)	49.6 (11.3)	53.7 (8.1)	50.0 (14.5)
Missing	10	4	2	1
Sex, n (%)				
Men	252 (90.3)	7 (100.0)	6 (100.0)	3 (100.0)
Women	27 (9.7)	0 (0.0)	0 (0.0)	0 (0.0)
Missing	10	4	2	1
Antihypertensive drug (yes), n (%)	153 (52.9)	5 (71.4)	4 (66.7)	2 (66.7)
Missing	0	4	2	1
Salt intake (g/day), Mean (SD)	11.3 (2.0)	11.5 (0.8)	12.5 (2.3)	11.7 (0.1)
Missing	10	4	2	1
BMI (kg/m2), Mean (SD)	25.3 (4.0)	27.0 (4.0)	27.8 (4.1)	28.5 (2.6)
Missing	10	4	2	1
Behavior change intentions, Mean (SD)	2.7 (1.2)	2.0 (0.4)	2.3 (0.7)	2.5 (0.6)
SBP (mmHg), Mean (SD)	127.8 (12.4)	129.3 (8.5)	133.3 (12.4)	119.3 (5.5)
Missing	10	4	2	1
DBP (mmHg), Mean (SD)	81.3 (10.5)	83.6 (7.1)	84.0 (6.9)	84.0 (1.7)
Missing	10	4	2	1

Abbreviations: BMI, body mass index; SBP, systolic blood pressure; DBP, diastolic blood pressure.

© 2025 Sato K et al. *JAMA Network Open.*

eTable 2. Comparison of Baseline Characteristics of Participants With Missing Outcomes

Characteristic	Overall	Missing Outcomes			
		Salt intake	BMI	Behavior change intentions	SBP/DBP
	N = 289	N = 21	N = 13	N = 3	N = 161
Age (year), Mean (SD)	51.3 (8.3)	49.1 (7.0)	48.7 (7.6)	48.7 (7.6)	49.4 (9.0)
Missing	10	10	10	0	2
Sex, n (%)					
Men	252 (90.3)	11 (100.0)	3 (100.0)	3 (100.0)	147 (92.5)
Women	27 (9.7)	0 (0.0)	0 (0.0)	0 (0.0)	12 (7.5)
Missing	10	10	10	0	2
Antihypertensive drug (yes), n (%)	153 (52.9)	5 (23.8)	1 (7.7)	1 (33.3)	76 (47.2)
Salt intake (g/day), Mean (SD)	11.3 (2.0)	12.5 (2.2)	13.1 (1.7)	13.1 (1.7)	11.4 (2.1)
Missing	10	10	10	0	2
BMI (kg/m2), Mean (SD)	25.3 (4.0)	27.5 (4.7)	25.2 (0.9)	25.2 (0.9)	24.9 (3.9)
Missing	10	10	10	0	2
Behavior change intentions, Mean (SD)	2.7 (1.2)	3.0 (1.2)	2.8 (1.1)	2.7 (0.6)	2.7 (1.2)
SBP (mmHg), Mean (SD)	127.8 (12.4)	126.5 (15.0)	137.3 (10.1)	137.3 (10.1)	126.3 (12.1)
Missing	10	10	10	0	2
DBP (mmHg), Mean (SD)	81.3 (10.5)	82.3 (7.5)	85.7 (2.5)	85.7 (2.5)	80.3 (9.2)
Missing	10	10	10	0	2

Abbreviations: BMI, body mass index; SBP, systolic blood pressure; DBP, diastolic blood pressure.

© 2025 Sato K et al. *JAMA Network Open.*

eTable3. Baseline Adjusted Mean Scores and Between-Group Differences in Outcomes at 3 Months

Outcome	n	Mean (SE)			Differences (95% CI)	
		Treatment	Control	App-Only	Treatment vs Control	Treatment vs App-Only
Salt intake (g/day)	268	11.1 (0.2)	11.3 (0.2)	11.0 (0.3)	-0.18 (-0.53 to 0.17)	0.11 (-0.51 to 0.73)
BMI (kg/m^2)	276	25.4 (0.1)	25.5 (0.1)	25.5 (0.3)	-0.01 (-0.31 to 0.29)	-0.05 (-0.59 to 0.49)
Behavior change intentions	276	3.0 (0.1)	2.8 (0.1)	3.2 (0.2)	0.14 (-0.12 to 0.40)	-0.20 (-0.66 to 0.26)
SBP (mmHg)	120	129.6 (2.5)	134.1 (3.0)	136.4 (4.3)	-4.46 (-11.04 to 2.13)	-6.80 (-16.52 to 2.91)
DBP (mmHg)	120	81.8 (1.8)	85.2 (2.1)	91.0 (3.0)	-3.37 (-8.00 to 1.27)	-9.19 (-16.04 to -2.35)

Abbreviations: BMI, body mass index; SBP, systolic blood pressure; DBP, diastolic blood pressure.
All models were adjusted for age, sex, antihypertensive medication use, salt intake, BMI, behavior change intentions, SBP, and DBP at baseline. Standard errors (SE) of a regression model were presented in the parentheses.

© 2025 Sato K et al. *JAMA Network Open.*

eMethods 5. Post Hoc Calculation of Minimum Detectable Effect Size (MDES)

$$MDES = \left(z_{(1-\alpha)/2} + z_{1-\beta}\right)\sigma\sqrt{\frac{1}{n_1} + \frac{1}{n_2}}$$

where the significance level α is set at .05, the observed standard deviation of salt intake σ is 2.0 g/day, and the sample sizes of the treatment and control groups n_1 and n_2 are 130 and 133, respectively.

If the statistical power $1 - \beta$ is set at .80,

$$MDES = (1.96 + 0.84) * 2.0 * \sqrt{\frac{1}{130} + \frac{1}{133}} = 0.69$$

If the statistical power $1 - \beta$ is set at .90,

$$MDES = (1.96 + 1.28) * 2.0 * \sqrt{\frac{1}{130} + \frac{1}{133}} = 0.80$$

© 2025 Sato K et al. *JAMA Network Open.*
