# Supplementary material for: Sodium Reduction Program Incorporating Genetic Profile and an AI-Based App: A Randomized Clinical Trial
Source: JAMA Netw Open. 2025 Oct 16;8(10):e2537540. doi: 10.1001/jamanetworkopen.2025.37540 (PMC12531883; doi:10.1001/jamanetworkopen.2025.37540)
Supplement: Supplement 3. — Data Sharing Statement [file jamanetwopen-e2537540-s003.pdf]

# Data Sharing Statement

Sato. Sodium Reduction Program Incorporating Genetic Profile and an AI-Based App. *JAMA Netw Open*. Published October 16, 2025. doi:10.1001/jamanetworkopen.2025.37540

**Data**
**Additional Information:** The University Hospital Medical Information Network Clinical Trials Registry (UMIN000052685); https://center6.umin.ac.jp/cgi-open-bin/ctr_e/ctr_view.cgi?recptno=R000060118
**Data available:** Yes
**Data types:** Deidentified participant data
**How to access data:** Data is available at the UMIN Individual Case Data Repository (https://center6.umin.ac.jp/ice/60118).
**When available:** With publication

**Supporting Documents**
**Document types:** None

**Additional Information**
**Who can access the data:** UMIN ID holders
**Types of analyses:** For any purpose
**Mechanisms of data availability:** Without investigator support
